# Serum Uric Acid and Triglycerides in Chinese Patients with Newly Diagnosed Moyamoya Disease: A Cross-Sectional Study

**DOI:** 10.1155/2019/9792412

**Published:** 2019-07-02

**Authors:** Wenyuan Ma, Changmeng Cui, Song Feng, Genhua Li, Guangkui Han, Yawei Hu, Xiang Li, Jianwei Lv, Chen Liu, Feng Jin

**Affiliations:** ^1^Clinical Medical College, Jining Medical University, Jining, Shandong 272067, China; ^2^Department of Neurosurgery, Affiliated Hospital of Jining Medical University & Shandong Provincial Key Laboratory of Stem Cells and Neuro-oncology, Jining, Shandong 272029, China

## Abstract

**Background:**

Evidence regarding the relationship between serum uric acid and triglycerides is limited. Therefore, the specific objective of this study was to investigate whether serum uric acid was independently related to triglycerides in Chinese patients with newly diagnosed moyamoya disease after adjusting for other covariates.

**Methods:**

The present study was a cross-sectional study. A total of 261 Chinese patients with newly diagnosed moyamoya disease were recruited from a hospital in China from 24 March 2013 to 24 December 2018. The independent variable and the dependent variable were serum uric acid measured at baseline and triglycerides, respectively. The covariates involved in this study included age, sex, body mass index, smoking status, and alcohol consumption.

**Results:**

The average age of the 227 selected participants was 47.5 ± 12.6 years old, and approximately 48.5% of them were male. The results of the fully adjusted linear regression showed that serum uric acid (10 *μ*mol/L) was positively associated with triglycerides (mmol/L) after adjusting for confounders (*β* 0.048, 95% CI 0.032, 0.064).

**Conclusions:**

In patients with moyamoya disease, there seemed to be a positive association between serum uric acid and triglycerides.

## 1. Introduction

Moyamoya disease (MMD) is a type of chronic cerebrovascular occlusion disease that frequently occurs in East Asian populations, including pediatric and adult patients, and may lead to ischemic or hemorrhagic stroke, headaches, epilepsy, or transient ischemic attack [1]. The major characteristic of MMD is a steno-occlusive change at the end of the internal carotid artery (ICA), middle cerebral artery (MCA), and/or proximal anterior cerebral artery (ACA) that is accompanied by the formation of smoke-like abnormal blood vessels at the base of the skull, as shown by digital subtraction angiography (DSA) [1, 2]. To date, the underlying mechanisms of MMD have remained to be fully elucidated. Existing studies on MMD have focused on its treatment and prognosis [3–6]. Nevertheless, evidence regarding the relationship between metabolic anomalies and MMD is limited [7].

Serum uric acid (SUA) is the final product of purine metabolism [8]. The relationship of SUA with cerebrovascular diseases is controversial [9–11]. Some studies have suggested that SUA has neuroprotective effects [9, 10]. However, some studies have shown that the level of SUA is a risk factor for cerebrovascular events [12]. A recent study confirmed that SUA is a risk factor for intracranial artery stenosis [13]. In addition, data from several studies suggest that triglycerides (TGs) are a risk factor for cerebrovascular diseases, including carotid stenosis and intracranial artery stenosis [14, 15]. Overall, SUA and TGs are extremely important in the pathological and physiological processes of cerebrovascular diseases. However, the relationship between SUA and TGs is complex and not fully elucidated [16].

MMD is a type of cerebrovascular disease characterized by chronic progressive steno-occlusion of the cerebral vessels. It should be noted that both SUA and TGs are associated with vascular stenosis. The present study aimed to investigate whether SUA was independently related to TGs in Chinese patients with newly diagnosed MMD. The relationship between SUA and TGs may be able to help predict the progressive steno-occlusion of the cerebral vessels that is associated with MMD and may be involved in the development of MMD.

## 2. Materials and Methods

### 2.1. Study Design

We conducted a cross-sectional study to address the relationship between SUA and TGs. The target independent variable was baseline SUA. The dependent variable was TGs.

### 2.2. Study Population

The data of Chinese patients with newly diagnosed MMD were nonselectively and consecutively collected from the Department of Neurosurgery, Affiliated Hospital of Jining Medical University, Jining, Shandong, China. Our data did not include identifiable participant data for the purpose of safeguarding patient privacy. Data were compiled from the hospital electronic medical record system. Participants' informed consent was not required in this study because of the retrospective nature of the cohort study. The hospital institutional review board approved this study.

The study initially collected data from a total of 261 participants. Participants' entry time and deadline for inclusion were 24 March 2013 and 24 December 2018, respectively. The clinical protocol for each participant was performed according to the Guidelines for Diagnosis and Treatment of Moyamoya Disease (Spontaneous Occlusion of the Circle of Willis) (2012 Edition) [17]. The diagnostic criteria were as follows: (i) Cerebral angiography must show at least the following findings: (1) stenosis or occlusion of the terminal portion of the intracranial ICA or proximal portions of the ACA and/or the MCA, (2) abnormal vascular networks in the vicinity of the occlusive or stenotic lesions in the arterial phase, and (3) bilaterality of the findings in (1) and (2). (ii) The following conditions must have been excluded: (1) atherosclerosis, (2) autoimmune disease, (3) meningitis, (4) brain tumors, (5) Down's syndrome, (6) von Recklinghausen's disease, (7) head injury, (8) cerebrovascular lesions after head irradiation, and (9) others. The inclusion criteria included patients hospitalized in our hospital who were newly diagnosed with MMD. Exclusion criteria included patients with myeloproliferative disorders who used cytotoxic drugs, pregnant women, lactating mothers, patients who were already taking diuretics or hypolipidemic medications, patients with renal or hepatic diseases and patients treated with antigout medications.

### 2.3. Variables

We obtained SUA and TGs at baseline and recorded them as continuous variables. The detailed process is described as follows: (1) After the patient was admitted to the hospital, in the fasting state, the peripheral venous blood was taken by the department nurse and quickly sent to the laboratory. (2) All the measurements were performed by the laboratory technician and the inspecting physician in our hospital laboratory.

The covariates used in this study can be classified as follows: (1) demographic data; (2) variables that have been reported by previous literature to affect SUA or TGs; and (3) those identified based on our clinical experiences. Therefore, the following variables were used to construct the fully adjusted model: (1) continuous variables: age and body mass index (BMI) (obtained at baseline); (2) categorical variables: sex, smoking status, and alcohol consumption (obtained at baseline).

### 2.4. Statistical Analysis

We present continuous variables in two ways. We express continuous variables with a normal distribution as the mean ± standard deviation, and we presented continuous variables with skewed distributions as medians (Q1-Q3). Categorical variables are expressed as frequencies or percentages. We used the *χ*^2^ test (categorical variables), the one-way ANOVA test (normal distribution), or the Kruskal-Wallis test (skewed distribution) to test for differences among different SUA groups (quartiles). The entire data analysis process can be divided into two steps. Step 1: Univariate and multivariate linear regression were employed. We constructed three models: model 1, no covariates were adjusted; model 2, only adjusted for sociodemographic data; and model 3, adjusted for the variables in model 2 as well as the covariates presented in Table 1. Step 2: To address the nonlinearity of SUA and TGs, a generalized additive model and smooth curve fitting (penalized spline method) were conducted. If nonlinearity was detected, we first calculated the inflection point using the recursive algorithm and then constructed a two-piecewise linear regression on both sides of the inflection point. We determined the best fit model based on the *P* values for the log likelihood ratio test. To ensure the robustness of the data analysis, we performed a sensitivity analysis. We converted SUA into a categorical variable and calculated the *P* for the trend. The purpose was to verify the results of SUA as a continuous variable and to observe the possibility of nonlinearity. All analyses were performed with the R statistical software packages (http://www.R-project.org, the R Foundation) and EmpowerStats (http://www.empowerstats.com, X&Y Solutions, Inc., Boston, MA).* P* values less than 0.05 (two-sided) were considered statistically significant.

## 3. Results

### 3.1. Baseline Characteristics of the Selected Participants

**Figure 1:**
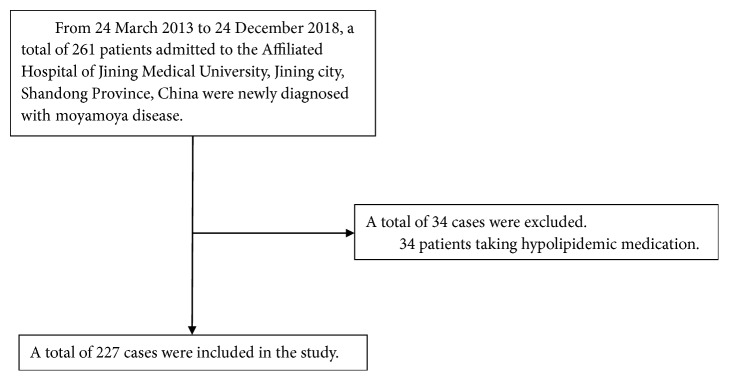
Inclusion/exclusion criteria.

A total of 227 participants were selected for the final data analysis based on the strict screening criteria (see inclusion and exclusion criteria for details) (see Figure 1 for a flow chart). The baseline characteristics of these selected participants are shown in Table 1 according to the quartile of SUA. The average age of the 227 selected participants was 47.5 ± 12.6 years, and approximately 48.5% of them were male. No statistically significant differences were detected in terms of age, BMI, total cholesterol (TC), low-density lipoprotein cholesterol (LDL-C), sex, smoking status, alcohol consumption, diabetes, or hypertension among the different SUA groups (all *P* values > 0.05). Participants in the group with the highest SUA (Q4) had higher TGs, very low-density lipoprotein cholesterol (VLDL-C), and SUA values than the participants in the other groups. The opposite patterns were observed for high-density lipoprotein cholesterol (HDL-C).

### 3.2. Univariate Analysis for TGs

We listed the results of the univariate analyses in Table 2. From the univariate linear regression, we found that sex, age, BMI, smoking status, alcohol consumption, and LDL-C were not associated with TGs. We also found that HDL-C (*β* -1.520, 95% CI -2.011, -1.029) was negatively associated with TGs. In contrast, univariate analysis showed that TC (*β* 0.190, 95% CI 0.048, 0.332), VLDL-C (*β* 1.855, 95% CI 1.599, 2.111), and SUA (*β* 0.049, 95% CI 0.036, 0.061) were positively correlated with TGs.

### 3.3. Results of Unadjusted and Adjusted Linear Regressions

In this study, we constructed three models to analyze the independent effects of SUA on TGs (univariate and multivariate linear regression). The effect sizes (*β*) and 95% confidence intervals are listed in Table 3. In the unadjusted model (model 1), the model-based effect size can be explained as an increase of 10 *μ*mol/L in SUA associated with TGs (mmol/L). For example, the effect size of 0.049 for TGs in the unadjusted model means that an increase of 10 *μ*mol/L in SUA is associated with a 0.049 mmol/L increase in TGs (mmol/L) (*β* 0.049, 95% CI 0.036, 0.061). In the minimum-adjusted model (model 2), when SUA increased by 10 *μ*mol/L, TGs (mmol/L) increased by 0.049 mmol/L (*β* 0.049, 95% CI 0.036, 0.062). In the fully adjusted model (model 3) (adjusted for all covariates presented in Table 1), for each additional 10 *μ*mol/L increase in SUA, TGs (mmol/L) increased by 0.048 mmol/L (*β* 0.048, 95% CI 0.032, 0.064). For the purpose of sensitivity analysis, we converted SUA from a continuous variable to a categorical variable (quartile of SUA), and the *P* for the trend of SUA as a categorical variable in the fully adjusted model was consistent with the result when SUA was a continuous variable.

### 3.4. The Results of the Association between SUA and TGs

**Figure 2:**
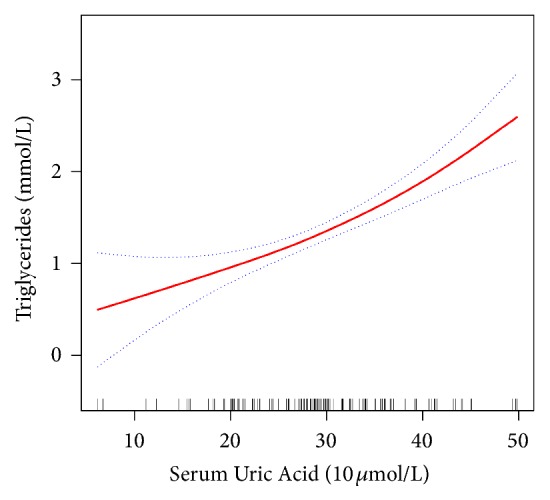
Association between SUA (10 *μ*mol/L) and TGs (mmol/L). The solid red line represents the smooth curve fit between the variables. Blue bands represent the 95% confidence interval of the fit. The model was adjusted for sex, age, BMI, smoking status, and alcohol consumption.

In the present study, we analyzed the relationship between SUA and TGs (Figure 2). The smooth curve and the result of the generalized additive model showed that the relationship between SUA and TGs was approximately linear after adjusting for age, sex, BMI, smoking status, and alcohol consumption.

## 4. Discussion

Our findings indicated that in Chinese patients with newly diagnosed MMD, SUA was positively associated with TGs after adjusting for other covariates. MMD, a rare cerebrovascular disorder of unknown etiology, is highly prevalent in East Asian countries and is associated with two main risks: cerebral ischemia and intracranial hemorrhage [17]. The major characteristic of MMD is a progressive occlusion change in the terminal portion of the ICA and its main branches, the ACA and the MCA [17, 18].

Elevated SUA concentration was significantly associated with proximal extracranial artery stenosis (PEAS) in the Chinese population [19]. A higher SUA level was associated with an increased risk of intracranial arterial stenosis (ICAS) among middle-aged females [20]. In addition, LDL-C is the principal target of lipid-lowering therapy to prevent carotid stenosis progression. However, even among patients with substantial reductions in LDL-C levels, residual cerebrovascular risk persists. High levels of TGs constitute an independent risk factor for the progression of carotid stenosis in patients with normal LDL-C levels [14]. Moreover, a study found that a history of a lipid disorder had the strongest association with the severity of intracranial stenosis [21]. Furthermore, our present study indicated that in Chinese patients with newly diagnosed MMD, SUA was positively associated with TGs. These findings showed that the underlying mechanisms between SUA levels and dyslipidemia may overlap. SUA promotes lipid peroxidation, produces oxy-radicals, and causes inflammation of the vascular wall. High levels of SUA are thought to be a mediator of proinflammatory endocrine disorders in adipose tissue, which may be one of the important factors involved in the development of dyslipidemia [22].

To the best of our knowledge, previous studies have rarely linked MMD with metabolic anomalies such as obesity and dyslipidemia. Nevertheless, a recent study showed a potential link between the pathogenesis of MMD and fat anomalies [23]. Apolipoprotein E (ApoE) in the cerebrospinal fluid of patients with MMD is downregulated. ApoE transports cholesterol and other lipids in the plasma and central nervous system by binding to cell-surface ApoE receptors. This process suggests that lipid metabolism plays a key role in the development and progression of MMD [24]. Ali N et al. [25] showed a significant positive relationship of SUA with TGs levels. Early prevention of hyperuricemia and dyslipidemia may be helpful to reduce the incidence of associated cardiovascular diseases. Similar findings were also reported in the study by Peng et al. [16]. Their conclusions are consistent with our findings.

The clinical value of this study is as follows. (1) To the best of our knowledge, this is the first study to observe the independent association between SUA and TGs in Chinese patients with newly diagnosed MMD. (2) The findings of this study could be helpful for future research on the establishment of diagnostic or predictive models for MMD. (3) The relationship between SUA and TGs may be able to help predict the progressive steno-occlusion of the cerebral vessels of patients with MMD and may be involved in the development of MMD.

Our study has some strengths. (1) We address nonlinearity in the present study, and (2) this study was an observational study and is therefore susceptible to potential confounding factors. We used strict statistical adjustments to minimize residual confounders.

There are some limitations in the present study: (1) In this study, our research subjects were Chinese patients with newly diagnosed MMD. Therefore, there is a certain deficiency in the universality and extrapolation of the research. In addition, due to the low incidence rate of MMD worldwide [1, 26], the sample size included in the present paper and other cross-sectional studies on MMD was relatively small [27, 28]. (2) Family history is also an important baseline characteristic for patients with MMD. However, among the data we collected in the present study, no patients had a family history of MMD. (3) Because we excluded patients with myeloproliferative disorders who were taking cytotoxic drugs, pregnant women, lactating mothers, patients who were already taking diuretic and hypolipidemic medications, patients with renal or hepatic diseases, and patients treated with antigout medications, the findings of this study cannot be generalized to these people.

## 5. Conclusions

In conclusion, the present study showed a positive association between SUA and TGs among Chinese patients with newly diagnosed MMD. This relationship may be involved in the pathogenesis of MMD.

## Figures and Tables

**Table 1:** Baseline characteristics of the participants.

SUA (10 *μ*mol/L, Min - Max)	Q1 (6.20-22.37)	Q2 (22.50-27.90)	Q3 (28.00-33.40)	Q4 (33.50-53.50)	*P*-value
Number	55	52	56	55	
Age (years, mean ± sd)	46.2 ± 12.5	47.4 ± 12.4	46.2 ± 13.2	49.4 ± 12.6	0.528
BMI (kg/m^2^, mean ± sd)	26.33 ± 4.07	25.67 ± 2.93	24.50 ± 4.05	25.68 ± 3.70	0.150
TGs (mmol/L, median, Q1 - Q3)	0.760 (0.600 - 1.050)	1.130 (0.788 - 1.353)	1.180 (0.910 - 1.658)	1.555 (1.302 - 2.230)	<0.001
TC (mmol/L, mean ± sd)	3.899 ± 0.916	4.030 ± 0.956	4.177 ± 0.798	4.363 ± 0.958	0.098
HDL-C (mmol/L, mean ± sd)	1.276 ± 0.270	1.198 ± 0.267	1.133 ± 0.218	1.090 ± 0.196	0.002
LDL-C (mmol/L, mean± sd)	2.153 ± 0.657	2.404 ± 0.786	2.415 ± 0.641	2.485 ± 0.722	0.135
VLDL-C (mmol/L, median, Q1 - Q3)	0.470 (0.225 - 0.590)	0.520 (0.375 - 0.685)	0.615 (0.408 - 0.845)	0.715 (0.458 - 0.955)	<0.001
SUA (10 *μ*mol/L, mean ± sd)	17.882 ± 3.888	25.261 ± 1.533	30.301 ± 1.641	39.573 ± 5.247	<0.001
Sex, n (%)					0.181
Female	27 (49.1%)	27 (51.9%)	23 (41.1%)	34 (61.8%)	
Male	28 (50.9%)	25 (48.1%)	33 (58.9%)	21 (38.2%)	
Smoking status, n (%)					0.387
NO	35 (66.0%)	38 (73.1%)	43 (76.8%)	44 (80.0%)	
YES	18 (34.0%)	14 (26.9%)	13 (23.2%)	11 (20.0%)	
Alcohol consumption, n (%)					0.517
NO	39 (73.6%)	39 (75.0%)	39 (69.6%)	45 (81.8%)	
YES	14 (26.4%)	13 (25.0%)	17 (30.4%)	10 (18.2%)	
Diabetes, n (%)					0.332
NO	52 (94.5%)	50 (96.2%)	49 (87.5%)	51 (92.7%)	
YES	3 (5.5%)	2 (3.8%)	7 (12.5%)	4 (7.3%)	
Hypertension, n (%)					0.501
NO	41 (74.5%)	33 (63.5%)	40 (71.4%)	35 (63.6%)	
YES	14 (25.5%)	19 (36.5%)	16 (28.6%)	20 (36.4%)	

Abbreviations. BMI: body mass index; TGs: triglycerides; TC: total cholesterol; HDL-C: high-density lipoprotein cholesterol; LDL-C: low-density lipoprotein cholesterol; VLDL-C: very low-density lipoprotein cholesterol; SUA: serum uric acid.

**Table 2:** Univariate analysis for TGs (mmol/L).

Covariate	Statistics	*β* (95% CI)	*P-*value
Sex			
Female	117 (51.5%)	Reference	
Male	110 (48.5%)	-0.096 (-0.362, 0.170)	0.481
Age, years	47.5 ± 12.6	-0.002 (-0.012, 0.009)	0.765
BMI, kg/m^2^	25.44 ± 3.78	-0.001 (-0.044, 0.042)	0.969
Smoking status			
NO	166 (73.8%)	Reference	
YES	59 (26.2%)	-0.188 (-0.486, 0.109)	0.216
Alcohol consumption			
NO	169 (75.1%)	Reference	
YES	56 (24.9%)	0.001 (-0.298, 0.300)	0.995
TC, mmol/L	4.127 ± 0.922	0.190 (0.048, 0.332)	0.010
HDL-C, mmol/L	1.174 ± 0.247	-1.520 (-2.011, -1.029)	<0.001
LDL-C, mmol/L	2.368 ± 0.709	0.029 (-0.160, 0.218)	0.762
VLDL-C, mmol/L	0.590 ± 0.352	1.855 (1.599, 2.111)	<0.001
SUA, 10 *μ*mol/L	28.305 ± 8.649	0.049 (0.036, 0.061)	<0.001

Abbreviations. BMI: body mass index; TC: total cholesterol; HDL-C: high-density lipoprotein cholesterol; LDL-C: low-density lipoprotein cholesterol; VLDL-C: very low-density lipoprotein cholesterol; SUA: serum uric acid; CI: confidence interval.

**Table 3:** Relationship between SUA (10 *μ*mol/L) and TGs (mmol/L) in different models.

Variable	Crude Model		Model I		Model II	
	*β* (95% CI)	*P*-value	*β* (95% CI)	*P*-value	*β* (95% CI)	*P*-value
SUA, 10 *μ*mol/L	0.049	<0.001	0.049	<0.001	0.048	<0.001
	(0.036, 0.061)		(0.036, 0.062)		(0.032, 0.064)	
SUA (quartile)						
Q1	Reference		Reference		Reference	
Q2	0.154	0.384	0.160	0.372	0.055	0.812
	(-0.191, 0.499)		(-0.190, 0.510)		(-0.394, 0.504)	
Q3	0.495	0.005	0.505	0.005	0.454	0.036
	(0.155, 0.835)		(0.158, 0.852)		(0.035, 0.873)	
Q4	0.996	<0.001	1.024	<0.001	0.911	<0.001
	(0.673, 1.320)		(0.690, 1.358)		(0.507, 1.314)	
*P* for the trend	<0.001		<0.001		<0.001	

Abbreviations. SUA: serum uric acid; CI: confidence interval.

Model I adjusted for sex and age.

Model II adjusted for sex, age, BMI, smoking status, and alcohol consumption.

## Data Availability

The data used to support the findings of this study are available from the corresponding author upon request.

## References

[1] Zhang H., Zheng L. (2019). Epidemiology, diagnosis and treatment of moyamoya disease. *Experimental and Therapeutic Medicine*.

[2] Lee S. U., Oh C. W., Kwon O. K. (2018). Surgical treatment of adult moyamoya disease. *Current Treatment Options in Neurology*.

[3] Kraemer M., Karakaya R., Matsushige T. (2018). Efficacy of STA–MCA bypass surgery in moyamoya angiopathy: long-term follow-up of the Caucasian Krupp Hospital cohort with 81 procedures. *Journal of Neurology*.

[4] Yuan J., Qu J., Zhang D. (2019). Cerebral perfusion territory changes after direct revascularization surgery in moyamoya disease: a territory arterial spin labeling study. *World Neurosurgery*.

[5] Zeifert P. D., Karzmark P., Bell-Stephens T. E., Steinberg G. K., Dorfman L. J. (2017). Neurocognitive performance after cerebral revascularization in adult moyamoya disease. *Stroke*.

[6] Ravina K., Rennert R. C., Strickland B. A., Chien M., Carey J. N., Russin J. J. (2018). Pedicled temporoparietal fascial flap for combined revascularization in adult moyamoya disease. *Journal of Neurosurgery*.

[7] Scott R. M., Smith E. R. (2009). Moyamoya disease and moyamoya syndrome. *The New England Journal of Medicine*.

[8] Logallo N., Naess H., Idicula T. T., Brogger J., Waje-Andreassen U., Thomassen L. (2011). Serum uri acid: neuroprotection in thrombolysis. The Bergen NORSTROKE study. *BMC Neurology*.

[9] Ya B., Liu Q., Li H. (2018). Uric acid protects against focal cerebral ischemia/reperfusion-induced oxidative stress via activating Nrf2 and regulating neurotrophic factor expression. *Oxidative Medicine and Cellular Longevity*.

[10] Zhang B., Yang N., Lin S., Zhang F. (2017). Suitable concentrations of uric acid can reduce cell death in models of OGD and cerebral ischemia–reperfusion injury. *Cellular and Molecular Neurobiology*.

[11] Sciacqua A., Perticone M., Tassone E. J. (2015). Uric acid is an independent predictor of cardiovascular events in post-menopausal women. *International Journal of Cardiology*.

[12] Li Z., Yi C., Li J., Tang N. (2017). Serum uric acid level as a cardio-cerebrovascular event risk factor in middle-aged and non-obese Chinese men. *Oncotarget*.

[13] Li M., Huang Y., Lin H., Chen Y. (2019). Association of uric acid with stenosis of intracranial and extracranial arteries in elderly patients with cerebral infarction. *Neurological Sciences*.

[14] Kitagami M., Yasuda R., Toma N. (2017). Impact of hypertriglyceridemia on carotid stenosis progression under normal low-density lipoprotein cholesterol levels. *Journal of Stroke and Cerebrovascular Diseases*.

[15] Zhang Z., Xiao M., Ye Z., Zhang W., Han B., Li Y. (2015). Noncardiogenic stroke patients with metabolic syndrome have more border-zone infarction and intracranial artery stenosis. *Journal of Stroke and Cerebrovascular Diseases*.

[16] Peng T.-C., Wang C.-C., Kao T.-W. (2015). Relationship between hyperuricemia and lipid profiles in US adults. *BioMed Research International*.

[17] Research Committee on the Pathology and Treatment of Spontaneous Occlusion of the Circle of Willis (2012). Guidelines for diagnosis and treatment of moyamoya disease (spontaneous occlusion of the circle of Willis). *Neurologia medico-chirurgica*.

[18] Yamamoto S., Kashiwazaki D., Uchino H. (2019). Stenosis severity-dependent shrinkage of posterior cerebral artery in moyamoya disease. *World Neurosurgery*.

[19] Yang X., Lv H., Hidru T. H. (2018). Relation of serum uric acid to asymptomatic proximal extracranial artery stenosis in a middle-aged Chinese population: a community-based cross-sectional study. *BMJ Open*.

[20] Ahn J., Hwang J., Hwang J., Yoon W., Chung P., Ryu S. (2018). The association between serum uric acid and asymptomatic intracranial arterial stenosis in middle-aged Koreans. *Nutrition, Metabolism & Cardiovascular Diseases*.

[21] Turan T. N., Makki A. A., Tsappidi S. (2010). Risk factors associated with severity and location of intracranial arterial stenosis. *Stroke*.

[22] Baldwin W., McRae S., Marek G. (2011). Hyperuricemia as a mediator of the proinflammatory endocrine imbalance in the adipose tissue in a murine model of the metabolic syndrome. *Diabetes*.

[23] Sugihara M., Morito D., Ainuki S. (2019). The AAA+ ATPase/ubiquitin ligase mysterin stabilizes cytoplasmic lipid droplets. *The Journal of Cell Biology*.

[24] Kashiwazaki D., Uchino H., Kuroda S. (2017). Downregulation of Apolipoprotein-E and apolipoprotein-J in moyamoya disease—a proteome analysis of cerebrospinal fluid. *Journal of Stroke and Cerebrovascular Diseases*.

[25] Ali N., Rahman S., Islam S. (2019). The relationship between serum uric acid and lipid profile in Bangladeshi adults. *BMC Cardiovascular Disorders*.

[26] Baba T., Houkin K., Kuroda S. (2008). Novel epidemiological features of moyamoya disease. *Journal of Neurology, Neurosurgery & Psychiatry*.

[27] Wang J., Chen G., Yang Y. (2018). Association between champagne bottle neck sign of internal carotid artery and ipsilateral hemorrhagic stroke in patients with moyamoya disease. *World Neurosurgery*.

[28] Funaki T., Takahashi J. C., Houkin K. (2018). Angiographic features of hemorrhagic moyamoya disease with high recurrence risk: a supplementary analysis of the Japan Adult Moyamoya Trial. *Journal of Neurosurgery*.

